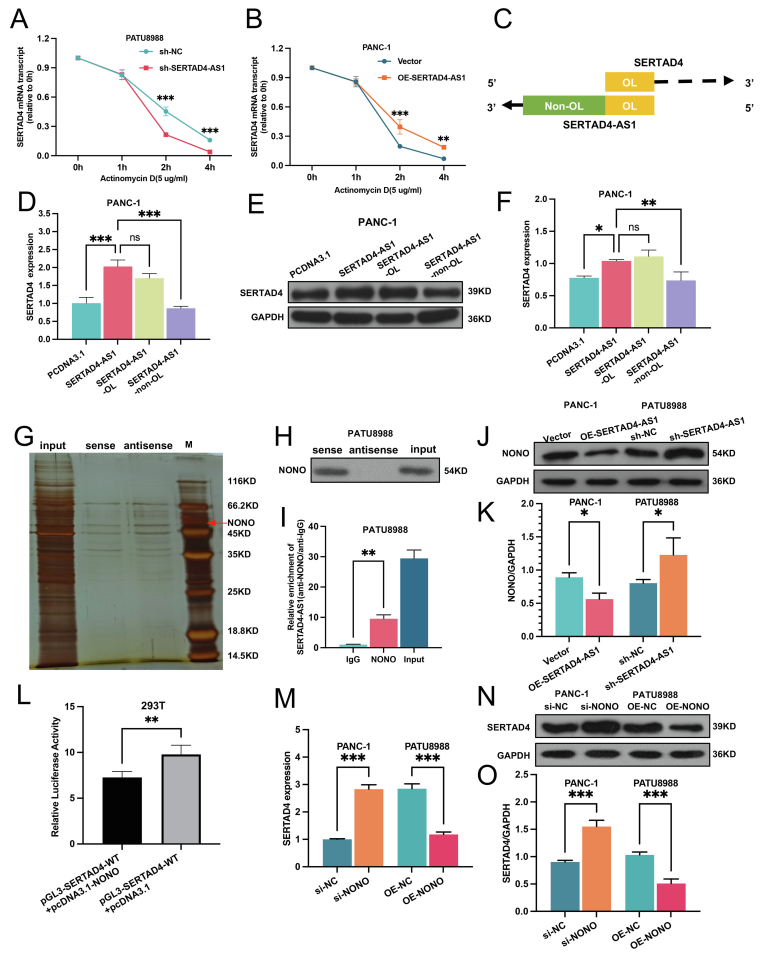# SERTAD4-AS1 suppresses pancreatic cancer progression by stabilizing SERTAD4 and inhibiting the Notch1 pathway

**DOI:** 10.1016/j.gendis.2025.101870

**Published:** 2025-09-23

**Authors:** Yuchen Tang, Jie Wang, Jun Yao, Haoran Li, Bin Yi, Chengqing Yu, Yijun Wei, Jian Yang, Zixiang Zhang, Jian Zhou

**Affiliations:** a Department of General Surgery, The First Affiliated Hospital of Soochow University, Suzhou, Jiangsu 215006, China; b Department of General Surgery, Suzhou Dushu Lake Hospital, The Fourth Affiliated Hospital of Soochow University, Suzhou, Jiangsu 215006, China; c State Key Laboratory of Radiation Medicine and Protection, Soochow University, Suzhou, Jiangsu 215123, China

Pancreatic cancer (PC) is a highly lethal malignancy with a poor prognosis.^1^ Long noncoding RNAs (lncRNAs), particularly natural antisense transcripts, play critical roles in regulating gene expression and tumorigenesis.^2^ Antisense lncRNAs modulate tumor progression by regulating the expression of their corresponding sense strands and activating downstream signaling pathways.^3^, ^4^, ^5^ However, the specific mechanism of their positive-sense action and the molecular mechanisms of their tumor-promoting or tumor-suppressive effects remain undefined. In this study, we demonstrated that SERTAD4-AS1 inhibited the proliferation and invasion of PC cells through the Notch1 signaling pathway. Mechanistically, SERTAD4-AS1 stabilized SERTAD4 mRNA by forming a double-stranded RNA structure with SERTAD4. Moreover, SERTAD4-AS1 directly bound to the NONO protein and prevented the transcriptional repression of SERTAD4 by NONO. Collectively, these results suggested the regulatory mechanism of SERTAD4-AS1 and uncovered the importance of the SERTAD4-AS1/NONO/SERTAD4/Notch1 pathway in PC. These findings also indicated that SERTAD4-AS1 may serve as a therapeutic target for PC treatment.

Kaplan–Meier survival analysis of SERTAD family members (SERTAD1, SERTAD2, SERTAD3, SERTAD4, and SERTAD4-AS1) revealed that lower expression levels of these genes were significantly correlated with poorer survival outcomes in PC patients. Notably, the prognostic significance of SERTAD4-AS1 was particularly pronounced in stage II patients, where reduced expression robustly predicted worse clinical outcomes (Fig. S1). Comprehensive pan-cancer expression profiling using the GENT2 database revealed significant down-regulation of SERTAD4-AS1 across 13 distinct malignancies, including pancreatic, bladder, breast, colon, kidney, and liver cancers (*p* < 0.001). These findings highlighted the potential relevance of SERTAD4-AS1 in oncogenesis (Fig. S2A). Validation in a clinical cohort of 50 paired patient samples (Table S1) from the First Affiliated Hospital of Soochow University via qRT-PCR confirmed markedly diminished SERTAD4-AS1 expression in pancreatic tumor tissues than in adjacent non-tumor (NT) tissues (*p* < 0.001) (Fig. S2B and S2C). In addition, the expression level of SERTAD4-AS1 was found to be significantly decreased in the T3 stage group (*p* < 0.01), the lymph node-positive group (*p* < 0.05) and the stage II group (*p* < 0.001), further underscoring its association with disease progression and aggressive tumor biology (Fig. S2D–S2G).

Functional characterization confirmed the tumor-suppressive role of SERTAD4-AS1 in PC cells. Overexpression of SERTAD4-AS1 in low-expressing PANC-1 cells significantly inhibited cellular proliferation, colony formation, migration, and invasion. Conversely, targeted knockdown of SERTAD4-AS1 in high-expressing PATU8988 cells consistently enhanced these malignant phenotypes, thereby establishing a direct functional link between SERTAD4-AS1 levels and oncogenic behaviors (Fig. S3). Subcellular localization analysis via RNA fluorescence in situ hybridization (RNA-FISH) demonstrated that SERTAD4-AS1 is present in both the nuclear and cytoplasmic compartments, suggesting its potential involvement in transcriptional and post-transcriptional regulation (Fig. S4A). To elucidate the underlying molecular mechanisms, a lncRNA–mRNA interaction network was constructed using the Co-LncRNA database, which computationally predicted potential interaction between SERTAD4-AS1 and the Notch1 signaling pathway (Fig. S4B). Subsequent Western blot analysis validated that overexpression of SERTAD4-AS1 significantly reduced Notch1 protein levels (Fig. S4C and S4D), concurrently suppressed the phosphorylation of the key signaling nodes mTOR and AKT (Fig. S4E and S4F), and elevated PTEN expression (Fig. S4G). Importantly, rescue experiments employing the specific Notch1 inhibitor Tangeritin effectively reversed the oncogenic effects, including signaling pathway hyperactivation, enhanced proliferation, and increased invasion, induced by SERTAD4-AS1 knockdown in PATU8988 cells (Fig. S7A–S7I), confirming its functional dependency on the Notch1 signaling pathway.

Further investigation revealed a critical relationship between SERTAD4-AS1 and its sense protein-coding gene, SERTAD4. Genomic analysis confirmed a partially overlapping region between SERTAD4-AS1 and the sense strand of SERTAD4 via the National Center of Biotechnology Information (NCBI) (Fig. S5A). Analysis of The Cancer Genome Atlas (TCGA) dataset via GEPIA2 demonstrated a significant positive correlation between their expression levels in PC tissues (*p* < 0.001) (Fig. S5B). QRT-PCR and Western blot analyses confirmed that SERTAD4-AS1 overexpression positively regulated SERTAD4 expression at both the mRNA and protein levels (Fig. S5C–S5E). Immunohistochemical (IHC) assessment of SERTAD4 protein expression in 44 PC tissues and 38 normal tissues clearly established its significant reduction in tumor samples (*p* < 0.001) (Fig. S5F and S5G). To further investigate the role of SERTAD4 in pancreatic cancer cells, we constructed lentivirus-mediated plasmids for the overexpression and knockdown of SERTAD4 and verified their transfection efficiency (Fig. S5H and S5I). Functionally, SERTAD4 itself exhibited tumor–suppressive properties: its overexpression in PANC-1 cells inhibited proliferation (Fig. S5J and S5K), colony formation (Fig. S5L and S5M), migration, and invasion (Fig. S6A–S6E) *in vitro*, and significantly reduced tumor volume and weight in a xenograft mouse model *in vivo* (Fig. S6F–S6I). Mechanistically, similar to SERTAD4-AS1, overexpression of SERTAD4 suppressed the Notch1 signaling pathway, as evidenced by reduced Notch1 protein levels (Fig. S6J and S6K), decreased mTOR and AKT phosphorylation (Fig. S6L and S6M), and altered PTEN expression (Fig. S6N). Importantly, these effects were also reversed by treatment with the specific Notch1 inhibitor, Tangeritin (Fig. S7J–S7R), further confirming SERTAD4's role as a regulator of the Notch1 signaling pathway in PC progression.

To further investigate whether SERTAD4-AS1 is dependent on SERTAD4, we co-transfected SERTAD4-AS1-overexpressing PANC-1 cells with si-SERTAD4 (Fig. S8A and S8B). Rescue experiments confirmed the functional dependence of SERTAD4-AS1 on SERTAD4. Co-transfection with si-SERTAD4 reversed the suppression of proliferation (Fig. S8C and S8D) and invasion (Fig. S8E and S8F) mediated by SERTAD4-AS1 overexpression. Furthermore, the suppressive effect of SERTAD4-AS1 overexpression on the Notch1 signaling pathway components (Notch1, p-mTOR, p-AKT) was also significantly reversed by concurrent SERTAD4 silencing (Fig. S8G–S8K), thereby demonstrating the functional interdependence between SERTAD4-AS1 and SERTAD4.

The study investigated the mechanisms by which SERTAD4-AS1 regulates SERTAD4 expression, revealing two complementary pathways. First, mRNA stability assays using the transcriptional inhibitor Actinomycin D demonstrated that SERTAD4-AS1 knockdown accelerated the decay of SERTAD4 mRNA (*p* < 0.001), while its overexpression stabilized it (*p* < 0.01) (Fig. S9A and S9B). Constructs expressing full-length SERTAD4-AS1, its overlapping (OL) region with SERTAD4, or the non-overlapping (non-OL) region were transfected into PANC-1 cells. These results demonstrated that the stabilizing effect critically depended on the OL region, as its absence (non-OL construct) resulted in significantly reduced SERTAD4 mRNA (*p* < 0.001) and protein (*p* < 0.01) expression compared to that of full-length SERTAD4-AS1 (Fig. S9C–S9F), indicating that lncRNA/mRNA duplex formation is essential for maintaining mRNA stability. Second, RNA pull-down assays coupled with mass spectrometry identified 13 proteins uniquely bound to SERTAD4-AS1 (Fig. S9G and S9H). Among these, the nuclear transcriptional regulator NONO (non-POU domain containing octamer binding) was selected for validation. RNA immunoprecipitation (RIP) assays confirmed significant enrichment of SERTAD4-AS1 in complexes precipitated with an anti-NONO antibody compared to control IgG (Fig. S9I). Western blot analysis showed that overexpression of SERTAD4-AS1 decreased NONO protein levels, whereas knockdown of SERTAD4-AS1 increased NONO protein levels, indicating a negative regulatory relationship between them (Fig. S9J and S9K). A dual-luciferase reporter assay demonstrated that NONO bound to and repressed SERTAD4 transcription (*p* < 0.01) (Fig. S9L). Functionally, the modulation of NONO expression confirmed its role as a transcriptional repressor of SERTAD4, significantly reducing both SERTAD4 mRNA (*p* < 0.001) and protein (*p* < 0.001) levels (Fig. S9M–S9O). Therefore, SERTAD4-AS1 enhances SERTAD4 expression not only by stabilizing its mRNA via the overlapping region but also by binding to and down-regulating the transcriptional repressor NONO, thereby alleviating transcriptional repression (Fig. 1).

**Figure 1:**
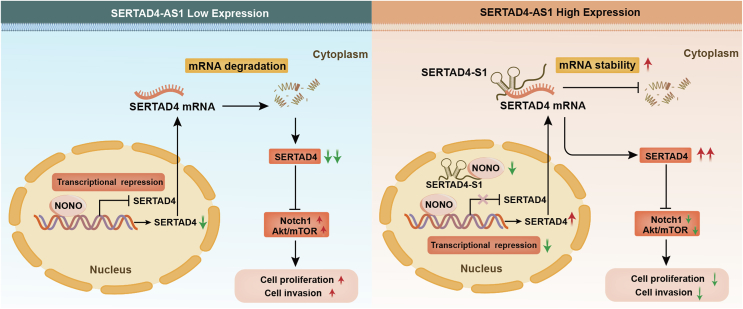
Mechanistic diagram of the SERTAD4-AS1/SERTAD4/Notch1 axis in pancreatic cancer. SERTAD4-AS1 alleviates the transcriptional suppression of SERTAD4 by interacting with NONO and consequently up-regulates SERTAD4 at the transcriptional level. SERTAD4-AS1 stabilizes SERTAD4 mRNA through its overlapping structure and protects it from degradation. The Notch1/AKT/mTOR signaling pathway is subsequently inhibited, and the proliferation and invasion of pancreatic cancer cells are attenuated.

Our identification of the SERTAD4-AS1/SERTAD4 axis as a critical tumor-suppressive pathway regulating the Notch1 signaling pathway revealed a complex regulatory mechanism in PC progression. This study elucidated a dual regulatory pathway where SERTAD4-AS1 stabilized SERTAD4 mRNA through direct lncRNA/mRNA duplex formation and alleviated transcriptional repression of SERTAD4 by binding to NONO, thereby inhibiting PC cell proliferation and invasion via SERTAD4-dependent attenuation of oncogenic Notch1/AKT/mTOR signaling. Thus, our findings uncovered a novel lncRNA-driven regulatory pathway that governs Notch1 activity and plays a critical role in PC progression. The consistent down-regulation of SERTAD4-AS1 in PC, along with its poor prognosis, underscores its clinical significance. Targeting the SERTAD4-AS1/NONO/SERTAD4 axis or its downstream effectors, such as the Notch1 signaling pathway, represents a promising therapeutic strategy for PC.

## CRediT authorship contribution statement

**Yuchen Tang:** Writing – original draft, Formal analysis, Data curation, Conceptualization. **Jie Wang:** Project administration, Methodology, Formal analysis. **Jun Yao:** Investigation, Formal analysis, Conceptualization. **Haoran Li:** Validation, Resources, Project administration. **Bin Yi:** Funding acquisition. **Chengqing Yu:** Supervision, Software, Methodology. **Yijun Wei:** Validation, Investigation, Data curation. **Jian Yang:** Writing – review & editing, Supervision, Funding acquisition, Formal analysis. **Zixiang Zhang:** Supervision, Resources, Project administration, Conceptualization. **Jian Zhou:** Writing – review & editing, Supervision, Funding acquisition, Conceptualization.

## Ethics approval and consent to participate

The study was approved by the Ethics Committee of the First Affiliated Hospital of Soochow University (Permit No. 2022407) and performed in strict accordance with the principles outlined in the Declaration of Helsinki. All animal experiments were approved by the Animal Protection Committee of Soochow University and complied with the relevant ethical regulations for animal research.

## Data availability

The datasets used during the current study are available from the corresponding author upon reasonable request.

## Funding

10.13039/501100001809National Natural Science Foundation of China (No. 82302921), Horizontal Project of Soochow University (China) (No. H231166), Suzhou Science and Technology Bureau Medical Health Technology Innovation Project (China) (No. SKYD2022106), Grant of State Key Laboratory of Radiation Medicine and Protection, Soochow University (China) (No. GZK12023037), Medical Applied and Basic Research Foundation of Suzhou Science & Technology Bureau (China) (No. SKY2023156), and Grant of the First Hospital of Soochow University Natural Science Foundation Incubation Program for Doctoral Trainees (China) (No. BXQN202218).

## Conflict of interests

The authors declared no competing interests.

## Figures and Tables

**figs1:**
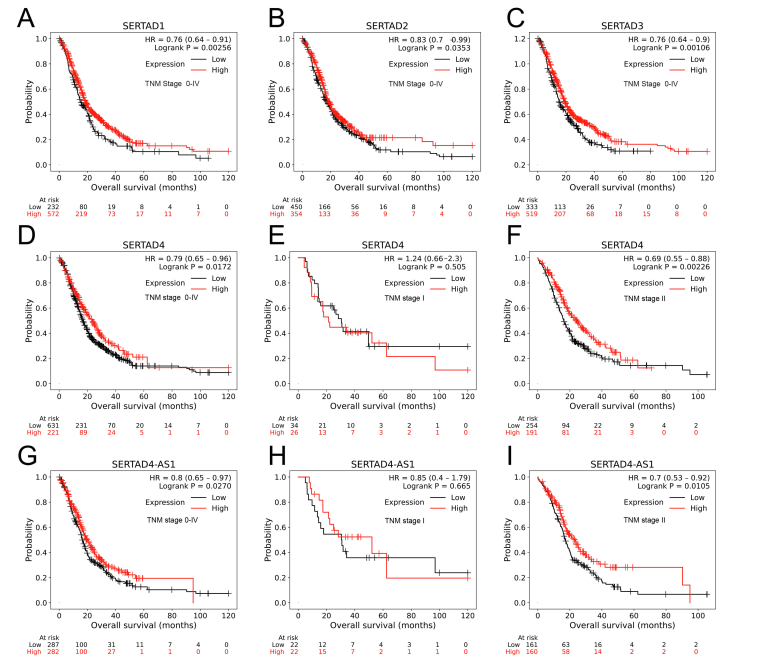


**figs2:**
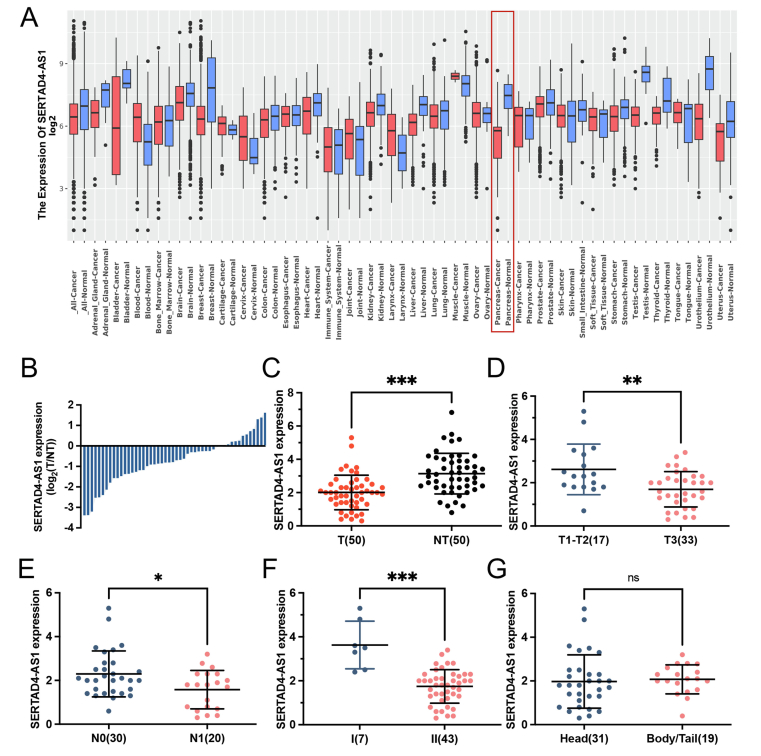


**figs3:**
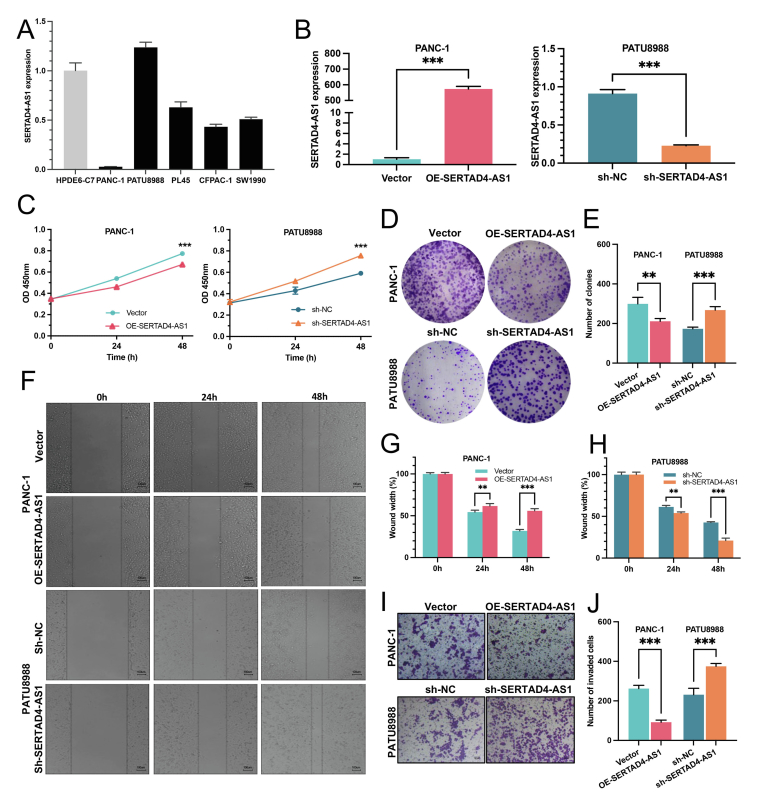


**figs4:**
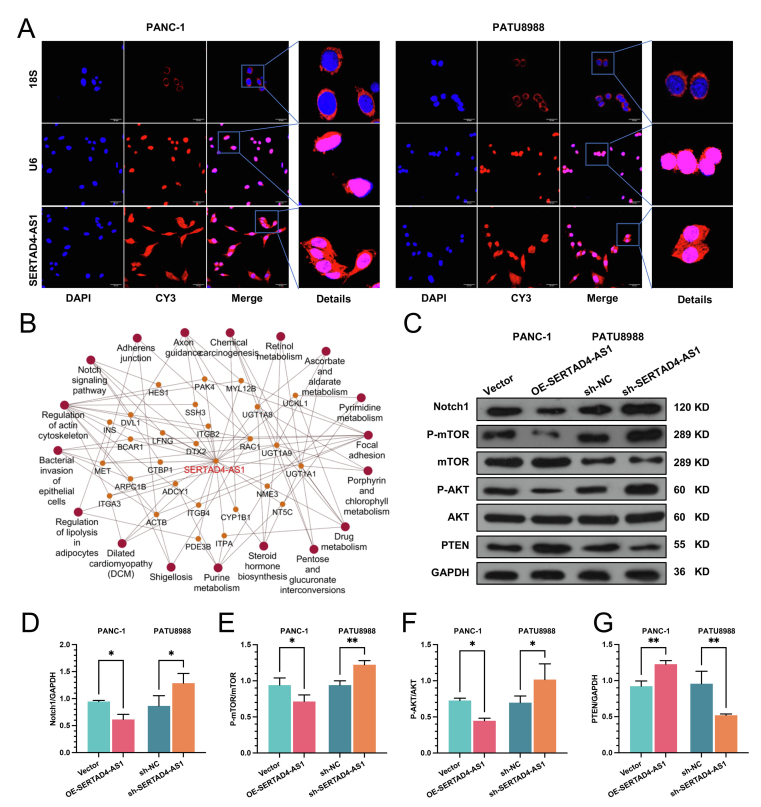


**figs5:**
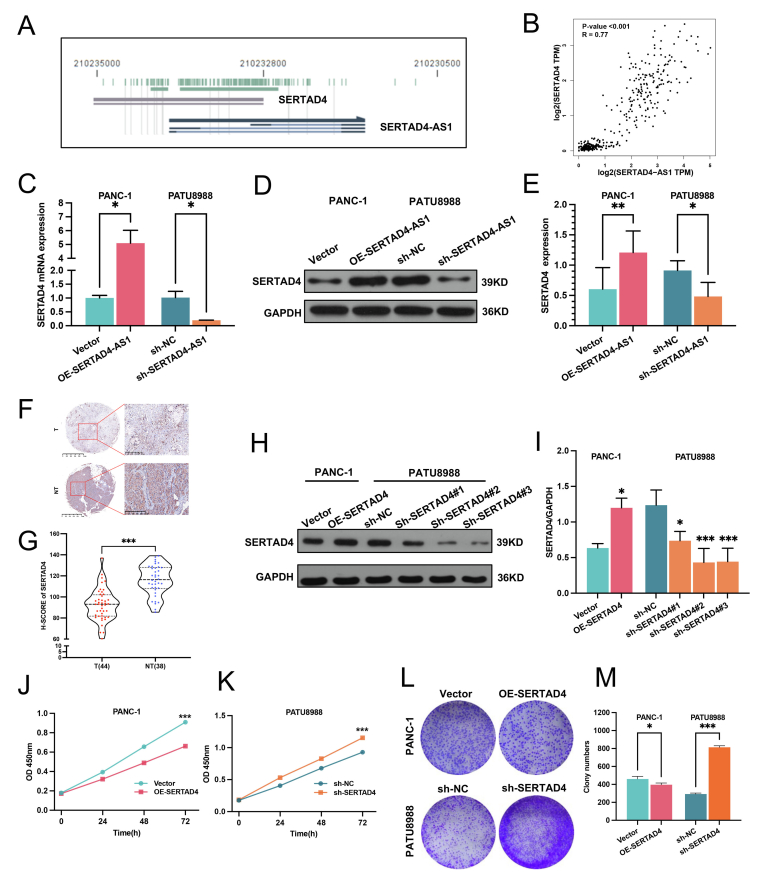


**figs6:**
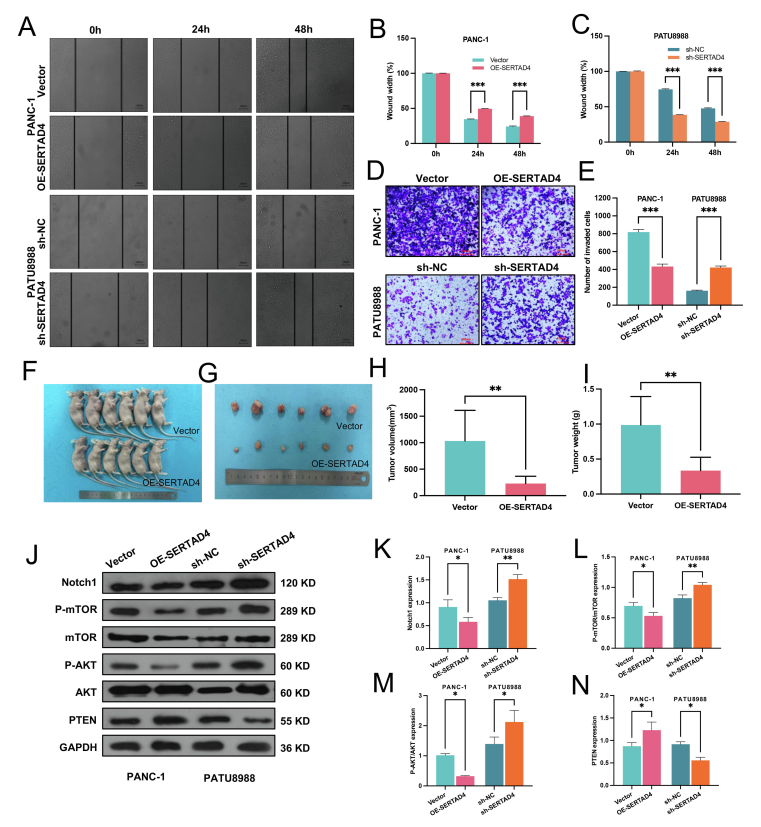


**figs7:**
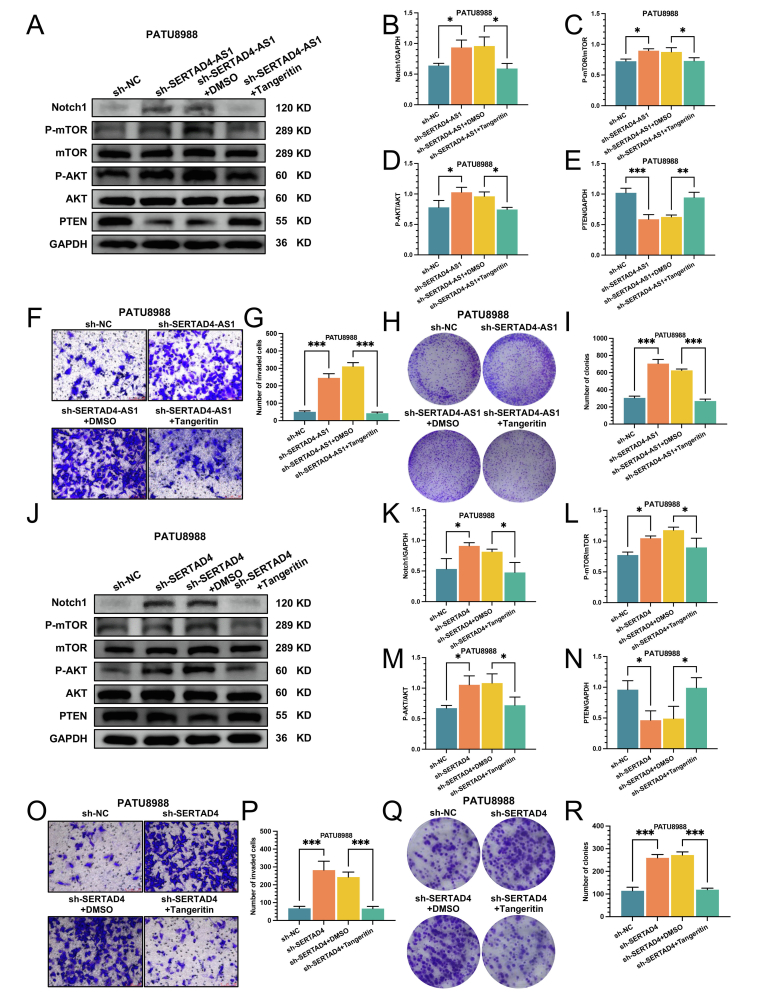


**figs8:**
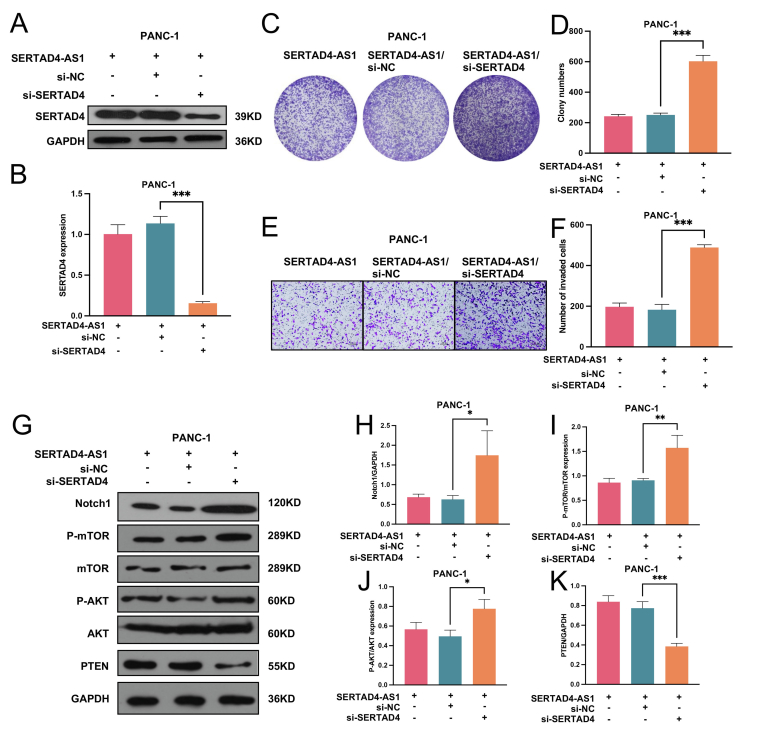


**figs9:**