# Supplementary material for: SERTAD4-AS1 suppresses pancreatic cancer progression by stabilizing SERTAD4 and inhibiting the Notch1 pathway
Source: Genes Dis. 2025 Sep 23;13(5):101870. doi: 10.1016/j.gendis.2025.101870 (PMC13123480; doi:10.1016/j.gendis.2025.101870)
Supplement: Multimedia component 1 [file mmc1.docx]

**Materials and methods**

**Clinical samples**

A total of 50 pairs of tumor (T) tissues and paired non-tumor (NT) pancreatic tissues were obtained from pancreatic cancer patients who underwent surgical treatment at the First Affiliated Hospital of Soochow University between 2016 and 2019. Fresh tissue samples were preserved using liquid nitrogen (50 pairs) or formalin (44 T tissues and 38 NT tissues), and the corresponding clinicopathological data are shown in Table S1. None of the patients underwent chemotherapy or radiotherapy before surgery and were regularly followed up after the operation.The study received approval from the Ethics Committee of the First Affiliated Hospital of Soochow University and was performed in strict accordance with the principles outlined in the Declaration of Helsinki.

**Cell lines and transfection**

Five pancreatic cancer cell lines (PANC-1, PATU8988, PL45, CFPAC-1, and SW1990) and one normal pancreatic cell line (HPDE6-C7) were obtained from the Shanghai Cell Bank of the Chinese Academy of Sciences (Shanghai, China). Small interfering RNAs (siRNAs) against human SERTAD4 were transfected using Lipofectamine 2000 (Invitrogen, USA), while nonspecific siRNA was used as a negative control. Small hairpin RNAs (shRNAs) targeting SERTAD4-AS1 and SERTAD4 lentivirus were purchased from GenePharma. Full-length lncRNA-SERTAD4-AS1 and SERTAD4 were synthesized, cloned, and inserted into lentiviral vectors, and an empty lentiviral vector was used as a control (GenePharma, China).

**Quantitative real-time PCR (qRT-PCR)**

TRIzol (Invitrogen, USA) was used to isolate total RNA from pancreatic cancer clinical samples or cells, in accordance with the manufacturer’s instructions, followed by separate concentration assessments. qRT-PCR was performed using SYBR Green I (TaKaRa, Japan) and LightCycler 480 (Roche, Germany) systems, with β-actin as the internal reference gene.

**Cell Counting Kit-8 (CCK-8) assay**

Cell viability was determined using the CCK-8 assay (Dojindo, Kumamoto, Japan). Cells were seeded in 96-well plates ( 1×10^4^-1×10^5^ cells/ well), followed by the addition of 10 μL of CCK-8 solution to each well. The IC50 of Tangeritin (MCE, USA) was determined using the CCK-8 assay, with an appropriate concentration of 15 μg/ mL. A microplate spectrophotometer (Thermo Fisher, USA) was used to evaluate the cell proliferation viability by calculating the absorbance at 450 nm.

**Colony formation assay**

Forty-eight hours post-transfection, cells were seeded in 6-well plates at a density of 1×10^3^ cells/well and cultured for 2 weeks. Cell colonies were then fixed with 4% formaldehyde and stained with Giemsa. Clones containing more than 50 cells were considered positive clones.

**Cell invasion assay**

The cell invasion assay was performed using BioCoat Matrigel Invasion Chambers (Corning, USA). Transfected cells were resuspended in serum-free medium at a density of 2 × 10^5^ cells/mL. Subsequently, 200 µL of the cell suspension was added to the upper chamber, while the lower chamber was filled with 500 µL of Dulbecco's Modified Eagle Medium (DMEM) supplemented with 10% fetal bovine serum. After incubation for 24 hours, invasive cells that had migrated to the lower chamber were fixed with 4% formaldehyde and stained with crystal violet. Finally, microscopic analysis and quantification were conducted by randomly selecting at least three independent fields of view.

**Cell migration assay**

Cell migration was evaluated using a wound healing assay. Specifically, a cell wound was created by scratching the center of the Petri dish with a 20 μL sterile pipette tip. The wound was then incubated in serum-free medium for 48 hours. Images of wound closure were taken every 24 hours and the change in the wound area was calculated.

**Western blotting**

Cells were harvested and lysed in RIPA lysis buffer (Beyotime Biotechnology, Shanghai, China) supplemented with protease and phosphate inhibitors for total protein extraction. Equal amounts of proteins were separated by 10% sodium dodecyl sulfate -polyacrylamide gel electrophoresis (SDS-PAGE) and transferred to a polyvinylidene fluoride membrane. The membrane was blocked with 5% skim milk and subsequently incubated overnight at 4 °C with the primary antibody, which was diluted according to the manufacturer's instructions in Tris-Buffered Saline and Tween (TBST) supplemented with 5% skim milk. The following day, the secondary antibody was prepared at the recommended concentration and incubated with the polyvinylidene fluoride (PVDF) membranes. Immunoreactive signals were detected using an enhanced chemiluminescence (ECL) detection system (FDbio, Shanghai, China), with GAPDH serving as an internal reference.

**Fluorescence in situ hybridization (FISH) assay**

PANC-1 and PATU8988 cells were plated onto culture slides, fixed with 4% paraformaldehyde (PFA), covered with prehybridization buffer, and incubated at 37 °C for 30 minutes. FISH probes labeled with CY3 were introduced to the mixture and then hybridized overnight at 37 °C. Following a 30-minute incubation with 4',6-diamidino-2-phenylindole (DAPI), high-resolution images of the cells were captured using a laser confocal microscope (Olympus-FV1200, Tokyo, Japan).

**LncRNA-mRNA interaction network**

The Co-LncRNA database (http://bio-bigdata.hrbmu.edu.cn/Co-LncRNA/) was used to identify mRNAs co-expressed with SERTAD4-AS1. Interaction networks were analyzed using Cytoscape 3.10.1 (https://cytoscape.org).

**Immunohistochemistry**

Formalin-fixed pancreatic tumor tissues and NT pancreatic tissues were embedded in paraffin, sectioned (4 µm), dehydrated, deparaffinized, and hydrated, as previously described. The slides were incubated overnight with SERTAD4 antibody (ab170259, Abcam, USA) in a dark room at 4 °C. DAB was used to stain the immune complexes, with positivity indicated by a brown color. Hematoxylin was used for nuclear counterstaining, with positivity indicated by blue color. Ultimately, the expression of proteins was evaluated through microscopic analysis.

H-score = Σ(pi × i) = (proportion of cells exhibiting weak staining intensity × 1) + (proportion of cells with moderate staining intensity × 2) + (proportion of cells displaying strong staining intensity × 3).

**RNA stability analysis**

PATU8988 and PANC-1 cells were treated with the transcriptional inhibitor actinomycin D (Sigma-Aldrich, USA) for 0, 1, 2, or 4 hours. To extract RNA, cells were lysed at different time points. Finally, qRT-PCR was performed to evaluate the mRNA expression levels of SERTAD4.

**RNA pull-down assay**

SERTAD4-AS1 was constructed as an overexpression plasmid. The T7 promoter sequence was incorporated in front of the forward primer, while the reverse primer sequence remained unchanged. The overexpression plasmid was used as a template and amplified using synthesized primers to generate an in vitro transcription template. The amplified PCR products were detected using agarose gel electrophoresis. The RNA obtained from in vitro transcription was labeled with biotin and purified using a MEGAclear^TM^ Transcription Clean-Up Kit (Thermo Fisher, USA). The resulting RNA-bound protein mixture was subjected to SDS-PAGE, followed by silver staining with a Pierce Silver Stain Kit (Thermo Fisher, USA). Specific binding protein bands were excised for subsequent mass spectrometry analysis. Raw mass spectrometry files were qualitatively and quantitatively analyzed using the UniProt Human database with Proteome Discoverer (version 2.4, Thermo Fisher, USA).

**RNA immunoprecipitation (RIP) assay**

A Magna RIP Kit (Millipore, GER) was used to analyze the association of NONO with SERTAD4-AS1. In brief, 2 × 10^7^ PATU8988 cell lysates were incubated with beads conjugated with NONO (Ptgcn, CHN) and negative control (normal mouse IgG). The immunoprecipitated RNAs were extracted. qRT–PCR was used to quantify the co-precipitated RNAs. The RIP assay was independently repeated three times to ensure reproducibility.

**In vivo experiment**

Animal experiments were approved by the Institutional Animal Conservation and Utilization Committee (IACUC) of Soochow University. Four-week-old BALB/c nude mice were obtained from Shanghai Experimental Animal Center (Shanghai, China). Subsequently, the mice were randomly allocated to two groups with six mice per group. OE-SERTAD4 and its corresponding vector were transfected into PANC-1 cells using lentiviral vectors. Subsequently, 5×10^6^ cells were slowly introduced into the subcutaneous tissue of the right flank of each nude mouse. Mice weight and tumor volume were measured at three-day intervals. Mice were euthanized on day 30 or when they reached the point of imminent death. Subsequently, the tumors were surgically excised, and their lengths and widths were measured to determine their volumes. The tumor volume was calculated using the following formula: tumor volume = length × width^2^ × π/6.

**Dual-luciferase reporter assay**

The pGL3 basic vector was used as a vector for firefly luciferase and renilla luciferase, and the promoter region of SERTAD4 was inserted into the 5' end of the dual luciferase reporter gene vector. Positive clones were screened, the reporter gene plasmid was amplified and purified for backup, and the transcription factor plasmid was amplified. 293T cells were selected for culture, and the reporter gene and transcription factor plasmids were co-transfected. Luciferin substrate was added to the samples, and the fluorescence intensity was measured using a fluorometer. Renilla luciferase was used as an internal control to exclude the effects of cell number and transfection efficiency.

**Statistical analysis**

Statistical analyses were performed with GraphPad Prism 9. Data are represented as the mean ± SD and were analyzed using a two-tailed Student’s t-test or one-way ANOVA. Survival differences among groups were estimated and compared by applying the Kaplan-Meier method along with the log-rank test. A P value below 0.05 was considered to indicate statistical significance.
