# Supplementary material for: SERTAD4-AS1 suppresses pancreatic cancer progression by stabilizing SERTAD4 and inhibiting the Notch1 pathway
Source: Genes Dis. 2025 Sep 23;13(5):101870. doi: 10.1016/j.gendis.2025.101870 (PMC13123480; doi:10.1016/j.gendis.2025.101870)
Supplement: Multimedia component 2 [file mmc2.docx]

**Figure S1** SERTAD4-AS1 and SERTAD4 expression associated with prognosis in pancreatic cancer patients. **(A–D)** Pancreatic cancer patients expressing high levels of SERTAD1, SERTAD2, SERTAD3 and SERTAD4 have improved overall survival compared to those with low levels. **(E)** Relationship between SERTAD4 expression and overall survival in patients with stage I pancreatic cancer. **(F)** Relationship between SERTAD4 expression and overall survival in patients with stage II pancreatic cancer. **(G)** Pancreatic cancer patients with low SERTAD4-AS1 expression had worse overall survival than those with high expression. **(H)** Relationship between SERTAD4-AS1 expression and overall survival in patients with stage I pancreatic cancer. **(I)** Relationship between SERTAD4-AS1 expression and overall survival in patients with stage II pancreatic cancer.

**Figure S2** SERTAD4-AS1 expression in databases and clinical specimens. **(A)** SERTAD4-AS1 expression in 36 tumor tissues and corresponding normal tissues by GENT2. **(B, C)** SERTAD4-AS1 expression in pancreatic tumor (T) and non-tumor (NT) pancreatic tissues. **(D, F)** Differential expression of SERTAD4-AS1 in tumors of T-, N-, and TNM-stage. **(G)** Expression of SERTAD4-AS1 in the head and body/tail of the pancreas. * *P <* 0.05, ** *P <* 0.01, and *** *P <* 0.001. ns, not significant.

**Figure S3** SERTAD4-AS1 inhibits the proliferation, invasion, and migration of pancreatic cancer cells *in vitro*. **(A)** SERTAD4-AS1 expression in normal pancreatic cells (HPDE6-C7) and in five types of pancreatic cancer cells (PANC-1, PATU8988, PL-45, CFPAC-1, and SW1990). **(B)** The transfection efficiency was verified by qRT-PCR. **(C)** CCK-8 assay showed the proliferative capacity after SERTAD4-AS1 overexpression or knockdown. **(D, E)** Colony formation assays. **(F–H)** Migration was determined with wound healing assays in PANC-1 or PATU8988 cells. Scale bar, 100 μm. **(I, J)** Invasion was determined with transwell assay in PANC-1 or PATU8988 cells. Scale bar, 50 μm. * *P <* 0.05, ** *P <* 0.01, and *** *P <* 0.001.

**Figure S4** SERTAD4-AS1 regulates the Notch1 signaling pathway.

**(A)** The results of the FISH experiments were observed via laser confocal microscopy. 18S and U6 are used as internal references. Red: Cy3 labels showing probe-labelled long non-coding RNA and internal reference; Blue: DAPI staining showing the cell nucleus. Scale bar, 30 μm. **(B)** SERTAD4-AS1 interaction-related genes and pathways predicted by bioinformatics analysis. **(C–G)** Regulation of the Notch1, AKT/mTOR and PTEN proteins by SERTAD4-AS1, as detected by Western blot. * *P <* 0.05, ** *P <* 0.01, and *** *P <* 0.001.

**Figure S5** SERTAD4-AS1 can positively regulate SERTAD4 expression.

**(A)** The relationship between SERTAD4-AS1 and SERTAD4. **(B)** Correlation between SERTAD4-AS1 and SERTAD4 expression analyzed via GEPIA2. **(C)** Expression of SERTAD4 after the overexpression or knockdown of SERTAD4-AS1 via qRT-PCR. **(D, E)** Expression of SERTAD4 after the overexpression or knockdown of SERTAD4-AS1 via Western blot. **(F, G)** Expression of SERTAD4 in tumor (T) and non-tumor (NT) tissues as determined by immunohistochemistry. Scale bar, 250 μm. **(H, I)** The efficiency of SERTAD4 overexpression or knockdown was evaluated by Western blot. **(J, K)** CCK-8 assay. **(L, M)** Colony formation assays. * *P <* 0.05, ** *P <* 0.01, and *** *P <* 0.001.

**Figure S6** SERTAD4 inhibits pancreatic cancer cell malignancy through the Notch1 signaling pathway. **(A–C)** Migration was determined with wound healing assays in PANC-1 or PATU8988 cells. Scale bar, 100 μm. **(D, E)** Invasion was determined with transwell assay. Scale bar, 100 μm. **(F–I)** Overexpression of SERTAD4 inhibits the volume and weight of subcutaneously transplanted tumors in nude mice. **(J–N)** Regulation of the Notch1, AKT/mTOR and PTEN proteins by SERTAD4, as detected by Western blot. * *P <* 0.05, ** *P <* 0.01, and *** *P <* 0.001.

**Figure S7** SERTAD4-AS1 and SERTAD4 inhibit pancreatic cancer cell malignancy through the Notch1 signaling pathway. **(A–E, J–N)** The protein expression of Notch1, AKT/mTOR, and PTEN in PATU8988 cells was detected via Western blot. **(F, G, O, P)** Transwell assays. Scale bar = 100 μm. **(H, I, Q, R)** Colony formation assays. * *P* < 0.05, ** *P <* 0.01, and *** *P* < 0.001.

**Figure S8** SERTAD4-AS1 can inhibit the proliferation and invasion of pancreatic cancer cells through SERTAD4. **(A, B)** PANC-1 cells were double-transfected with SERTAD4-AS1 and si-SERTAD4, and the protein expression of SERTAD4 was detected via Western blot. **(C, D)** Detection of the proliferative capacity of pancreatic cancer cells via colony formation analysis. **(E, F)** Invasive capacity of pancreatic cancer cells as assessed by the invasion assay. Scale bar, 500 μm. **(G–K)** The expression of Notch1, AKT/mTOR, and PTEN signaling pathway proteins was detected via Western blot. * *P <* 0.05, ** *P <* 0.01, and *** *P <* 0.001.

**Figure S9** The potential mechanisms by which SERTAD4-AS1 regulates SERTAD4.

**(A, B)** Stability of SERTAD4 mRNA at different time points as measured by qRT-PCR. **(C)** Schematic representation of the full-length, overlapping (OL), and non-overlapping (non-OL) structures of SERTAD4-AS1. **(D–F)** SERTAD4 expression after PANC-1 cells transfected with SERTAD4-AS1, SERTAD4-AS1-OL, or non-OL, as detected by qRT-PCR (D) and Western blot (E, F) analysis. **(G)** RNA pull-down and silver staining. **(H)** Direct binding of SERTAD4-AS1 to the NONO protein was detected via RNA pull-down. (I) RIP assays. **(J, K)** NONO expression after overexpression or knockdown of SERTAD4-AS1, as detected by Western blot. **(L)** Dual-luciferase reporter assay. **(M–O)** SERTAD4 expression was detected via qRT-PCR (M) and Western blot (N, O) analysis. * *P <* 0.05, ** *P <* 0.01, and *** *P <* 0.001.
