# Supplementary material for: SERTAD4-AS1 suppresses pancreatic cancer progression by stabilizing SERTAD4 and inhibiting the Notch1 pathway
Source: Genes Dis. 2025 Sep 23;13(5):101870. doi: 10.1016/j.gendis.2025.101870 (PMC13123480; doi:10.1016/j.gendis.2025.101870)
Supplement: Multimedia component 3 [file mmc3.docx]

**Supplementary Table S1：Clinicopathologic features of pancreatic cancer patients**

| Variables | Cases | SERTAD4-AS1 | |
| --- | --- | --- | --- |
|  |  | Low (23) | High (27) |
| Gender |  |  |  |
| Male | 30 | 14 | 16 |
| Female | 20 | 9 | 11 |
| Age（years） |  |  |  |
| ≥65 | 16 | 7 | 9 |
| <65 | 34 | 16 | 18 |
| Pathologic T |  |  |  |
| T1-T2 | 17 | 6 | 11 |
| T3 | 33 | 17 | 16 |
| Pathologic N |  |  |  |
| N0 | 30 | 10 | 20 |
| N1 | 20 | 13 | 7 |
| Pathologic stage (AJCC^8th^) |  |  |  |
| I | 7 | 0 | 7 |
| II | 43 | 23 | 20 |
| Location |  |  |  |
| Head | 31 | 17 | 14 |
| Body/Tail | 19 | 6 | 13 |
| Diabetes |  |  |  |
| Negative | 40 | 18 | 22 |
| Positive | 10 | 5 | 5 |
| Hypertension |  |  |  |
| Negative | 34 | 17 | 17 |
| Positive | 16 | 6 | 10 |
